# Emergency centre triage category allocations and their associated patient flow timeframes in a private healthcare group in the Middle East

**DOI:** 10.1002/nop2.336

**Published:** 2019-07-29

**Authors:** Enrico Dippenaar

**Affiliations:** ^1^ Division of Emergency Medicine University of Cape Town Cape Town South Africa; ^2^ Emergency Medicine Research Group Anglia Ruskin University Chelmsford UK

**Keywords:** emergency department, Middle East, nurses, time, triage

## Abstract

**Aims and Objectives:**

To identify and describe triage category allocations and their associated patient pathway timeframes in four emergency centres of a large private healthcare group in the United Arab Emirates.

**Background:**

The classification of patients in accordance with their acuity level is a complex task that requires quick and accurate allocation. Triage system categories have predetermined timeframes in which patients should be seen by a physician or treatment initiated for the best possible outcome.

**Design and Methods:**

An observational, cross‐sectional study was conducted through the prospective capture and evaluation of medical records from patients triaged in each of the four emergency centres (two hospitals and two clinics) over a period of a month. The STROBE statement was used as a reporting framework. Descriptive statistics were used to determine the timeframes associated with the patient pathway through each EC and contrasted against their allocated triage category.

**Results:**

A total of 4,432 patient records were eligible for analysis from the four emergency centres. Triage category 4 (54.7%) was allocated the most with only a single category 1 patient seen between the four emergency centres. The median time from registration to triage was <10 min and triage to physician consult was <25 min. The overall length of stay of high‐acuity cases was between 1 hr 13 min–2 hr 44 min, compared with low‐acuity cases being 32–49 min. Overall time to physician was substantially lower than the targets set by the triage systems itself.

## 1 INTRODUCTION

Triage plays an important role in the structure and organization of an emergency centre (EC) (Kennedy, Aghababian, Gans, & Lewis, 1996). The broad goal of triage is to identify the most critically ill or injured patients and to prioritize their timely care (Van der Linden, Lindeboom, Linden, & Lucas, 2012). The classification of patients related to their acuity level is a complex task that requires not only quick, but accurate allocation.

## 2 BACKGROUND

It is recognized internationally that triage should be conducted in a structured way that relies on the objective assessment of patients to determine their acuity based on medical evidence (Fry & Burr, 2002). This in theory should ensure that patients are stratified appropriately against the resources available in an EC. By allocating a higher triage category to higher acuity patients and vice versa, it allows an EC to structure its resources in a timely manner to attend to the most critically ill or injured first. The provision of timely care, especially in an EC, is one of the main goals of any triage system (Johnson, 1996). Its design is centred around timely allocation of resources that lead to timely patient treatment.

Although not perfect, triage systems are broken down into categories, usually ranging from three to five levels (Parenti, Manfredi, Bacchi Reggiani, Sangiorgi, & Lenzi, 2010). These categories are related to specified timeframes in which a patient should be seen by a physician or treatment initiated. The aim of this study was to identify and describe the timeframes associated with the patient pathway through four ECs of a large private healthcare group in the United Arab Emirates. This private hospital group uses a combination of five‐level triage systems in its ECs (Table 1). This study formed part of a larger research project that aimed to design and develop a standardized locally appropriate triage system (Dippenaar, 2016).

**Table 1:** Structural category differences of the four triage systems in use

Category	CTAS	MTS	ESI	SATS
1	Blue Resuscitation Immediate	Red Immediate Immediate	Level 1 Immediate	Red Emergency Immediate
2	Pink Emergent <15 min	Orange Very urgent <10 min	Level 2 High risk	Orange Very urgent <10 min
3	Yellow Urgent <30 min	Yellow Urgent <60 min	Level 3 Many different resources	Yellow Urgent <60 min
4	Green Less urgent <60 min	Green Standard <120 min	Level 4 One different resource	Green Routine <240 min
5	White Non‐urgent <120 min	Blue Non‐urgent <240 min	Level 5 No other resources	Blue Deceased

Abbreviations: CTAS, Canadian Triage and Acuity Scale; ESI, Emergency Severity Index; MTS, Manchester Triage System; SATS, South African Triage Scale.

## 3 METHODS

An observational, cross‐sectional study was conducted through the prospective capture and evaluation of patient medical records from the four ECs (two hospitals and two clinics) of the private hospital group in the Emirate of Dubai. The STROBE statement checklist (See File S1) for reporting observational studies was used as a framework (von Elm et al., 2008). The study received Research Ethics Committee approval from the hospital group and the University of Cape Town Human Research Ethics Committee in South Africa (HREC REF: 744/2014).

Medical records from patients triaged in each of the four ECs over a period of 1 month were evaluated and considered for inclusion. Electronic and manual platforms were used to collect the required data. The initial electronic data were sourced through the hospital groups’ medical records department at the end of the 1‐month period. EC staff were instructed to specifically include electronic data fields that was set out for this study, in addition to the usual EC data they capture. The electronically captured data from the four ECs during the month were collated and provided in a single Microsoft Excel (2016) spreadsheet. This included triage category allocations and patient flow timeframes (i.e., registration –> triage –> physician –> discharge). It was necessary to capture manual data that were not contained in the hospitals electronic information system. The manual data were captured by the triage nurses completing a one‐page form during their triage assessment of patients presenting to their ECs. Entries included the triage category allocation, time of triage, time of physician consult and time the patient leaves the EC. Internal training by the EC unit managers was conducted to familiarize the staff with the content of the data collection form. Medical record stickers with patient identifiers were attached to the form so that the data could later be merged with the electronic data. Clerical staff from the four ECs captured the manual data daily from the forms onto a custom spreadsheet. The researcher collected the spreadsheets from the four ECs and used the patient identifiers to merge the electronic and manual data sets through the merge data function. Patient identifiers (e.g., names, surnames and medical record numbers) were included in the data set shared with the researcher to provide an identifier for merging electronic data with manual data. Following this, all identifiers were stripped from the sample prior to analysis. The manual data capture forms were collected from the four ECs and handed back over to the hospital groups’ medical records department at the end of the study.

Only records with all the relevant data points were included. Records with missing data points were identified, filtered and removed from the database prior to analysis. Removing records from the data set may have introduced exclusion bias that could have resulted in the removal of potential outliers such as high‐acuity cases. Removing incomplete records before analysis ensured that a complete data set was available with all the data points present. There were no obvious reasons for data points to be missing other than random omission from the staff to make entries and thus obtaining these missing data points would not be possible.

The timeframes as patients moved through the ECs were captured at specific points in their journey from entering to leaving the EC by either being discharged or admitted to hospital. An observational analysis was done using non‐parametric descriptive statistics, with the median as a measure of central tendency as timeframes per triage category do not follow a normal distribution. Factors such as timestamp input delays, inaccurate time readings and adjusted time inputs that could have an impact on and alter these timeframes coupled with the uncertainty of their reliability and validity did not warrant in‐depth variance and relationship testing. Patient flow timeframes were contrasted against their allocated triage category, which in turn should have been guided by the triage systems themselves, as presented in Table 1. Unfortunately, the ECs were not using a single triage system exclusively at the time of this study, which made a direct comparison unrealistic. The proponent of this study was to identify current timeframes and match them with existing triage systems in the aim to create a standardized locally appropriate triage system, with realistic timeframe expectations.

## 4 RESULTS

**Figure 1:**
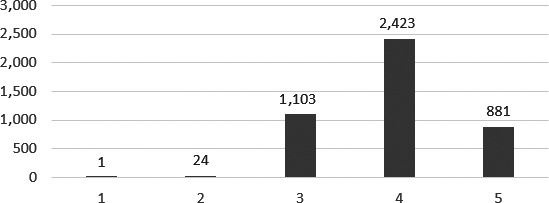
Triage category allocation from patient records (*N* = 4,432)

There were a total of 7,311 electronic and 6,754 manual patient records captured from the four ECs. When the data were combined in a single spreadsheet, there were some records captured electronically but not manually and vice versa, thus resulting in a smaller, combined number of 6,320 records. Duplicate and missing entries were removed leading to a further loss of 1,888 records and a final sample of 4,432 records. Of the 4,432 sampled records, triage category 4 was allocated most often (*N* = 2,423; 54.7%). Conversely, category 1 was only allocated once (Table 2 and Figure 1). Most of the allocations were made towards the mid‐ to low‐acuity spectrum (i.e., categories 3–5) (*N* = 4,407; 99.4%), whereas high‐acuity cases (categories 1 and 2) only made up an extremely small proportion of allocations (*N* = 25; 0.6%).

**Table 2:** Triage category allocation distribution from patient records

Category	Hospital ECs		Clinic ECs		Total	%
	EC1	EC2	EC3	EC4		
Total	2,333	1,199	496	404	4,432	
1	1				1	0.02
2	1	19	1	3	24	0.5
3	391	613	69	30	1,103	24.9
4	1,483	367	331	242	2,423	54.7
5	457	200	95	129	881	19.9

Of the 4,432 sampled records, there were only 2,997 records available for the timeframe: registration –> triage as some patients were directed straight to triage with registration occurring at a later stage. The other timeframes had all 4,432 sample records available. It was found that the overall median time from registration –> triage was <10 min (IQR 0–6 min) and registration –> physician consult was <20 min (IQR 0–19 min) (Table 3). The median triage –> consult times support the notion that patients were seen by a physician within 25 min (IQR 0–22 min) from the time they are triaged. EC1 was the only EC that saw a category 1 case; a physician saw them immediately. Category 2 cases were also seen immediately by physicians in all except EC2. They reported a median of 16 min (IQR 12–19 min). Timeframe data from EC2 showed a marked increase compared with the other ECs in the time it took for patients to be seen by a physician. In most cases, the median time was three to four times higher than the other ECs. The overall lengths of stay in the ECs were much longer for the mid‐ to high‐acuity cases (i.e., categories 1, 2 and 3) (IQR 1 hr 13 min–2 hr 44 min) with the lengths of stay of the low‐acuity cases (i.e., categories 4 and 5) (IQR 32 min–49 min) being markedly less. This decrease in lengths of stay of low‐acuity cases as compared with the mid‐ to high‐acuity cases is further evidenced by the decreased times from physician consult –> patients leaving the EC being 15–31 min.

**Table 3:** Patient flow timeframes (median & IQR) per triage category

Category	Hospital ECs		Clinic ECs	
	EC1	EC2	EC3	EC4
Registration at front desk → Triage at nurses' station (*N* = 2,997)				
1	0 (0–0)			
2	0 (0–0)	0 (0–1)	0 (0–0)	0 (0–0)
3	5 (2–11)	3 (1–7)	5 (3–10)	2 (0–6)
4	5 (2–10)	3 (1–6)	3 (1–8)	3 (1–7)
5	6 (3–11)	3 (1–7)	4 (2–11)	4 (2–8)
Triage at nurses' station → Consult with a physician (*N* = 4,432)				
1	0 (0–0)			
2	4 (4–4)	20 (11–28)	2 (1–3)	2 (1–3)
3	7 (4–12)	20 (12–30)	4 (2–5)	4 (3–5)
4	7 (4–12)	21 (13–33)	4 (3–5)	5 (3–7)
5	6 (4–13)	22 (12–33)	4 (3–7)	5 (3–8)
Consult with a physician → Patient leaves emergency centre (*N* = 4,432)				
1	90 (90–90)			
2	37 (37–37)	164 (118–209)	141 (141–141)	37 (36–88)
3	95 (49–140)	109 (63–150)	66 (26–105)	55 (33–96)
4	42 (17–90)	25 (15–51)	18 (11–40)	22 (15–41)
5	14 (10–26)	18 (12–31)	15 (11–28)	14 (11–18)
Triage at nurses' station → Patient leaves emergency centre (*N* = 4,432)				
1	90 (90–90)			
2	45 (45–45)	196 (137–232)	169 (169–169)	41 (39–91)
3	106 (59–154)	130 (87–173)	70 (37–114)	58 (38–99)
4	56 (27–102)	52 (36–83)	24 (16–44)	28 (21–45)
5	23 (16–42)	44 (31–63)	21 (15–38)	21 (16–29)

## 5 DISCUSSION

One of the most important validation criteria of any triage system is the time‐to‐physician variable (Beveridge et al., 1998; Gilboy, Tanabe, Travers, & Rosenau, 2011; Manchester Triage Group, 2006; South African Triage Group, 2012). The goal is to queue patients in such a manner that the larger patient numbers are appropriately coordinated to the smaller physician numbers or resources available. The triage systems in use in these ECs each have time targets set for patients to be seen. These time targets were mostly set arbitrarily by the creators of the triage systems based on reasonable expert opinion (i.e., not based on objective findings; Beveridge et al., 1998; Gilboy et al., 2011; Manchester Triage Group, 2006; South African Triage Group, 2012). They were, however, designed with the setting in mind that the triage systems would be used. Although there were observable differences in the overall timeframes of patients as they moved through the four ECs, especially from the two hospitals and two clinics, it was evident that the median time for patients from entering an EC to be seen by a physician was relatively short when compared with the timeframe targets of the existing triage systems (Beveridge et al., 1998; Gilboy et al., 2011; Manchester Triage Group, 2006; South African Triage Group, 2012). The time‐to‐physician times for categories 1 and 2 cases were in line with the set targets of the existing triage systems and would be very difficult to improve. Categories 3, 4 and 5, however, showed a marked decrease in time to physician as compared with the targets of existing triage systems. This suggested that the time‐to‐physician targets for all lower acuity cases could be made shorter, which would improve the overall waiting times. Overall, the inverse relationship between acuity level and time to physician and a direct relationship between the acuity levels and the length of stay in the EC are consistent across most ECs worldwide. In the private healthcare setting of this hospital group, the pressures of high‐acuity cases are substantially lower than those in the public sector (Dubai Health Authority, 2019; Health Authority Abu Dhabi, 2019). This decreased load allows for patients to be seen at a relatively fast pace, throughout all triage categories. It is noted, however, that EC2 had a markable increased time from triage to physician consult, even with half the patient load as compared with EC1. This could be due to the triage system, or a combination of systems they employ at that EC, or purely be an organizational issue in that EC that requires further investigation. EC1 was able to move higher acuity patients out of their EC quicker than the others, which may open available bed space as patient throughput is faster. This is a key element when evaluating the time‐to‐physician times in an EC. Being able to transfer patients out of an EC more readily allows for resources to be freed sooner, resulting in more patients that can be seen in a shorter period of time (Gravel et al., 2013; van der Wulp, 2010). This is especially true when considering this hospital group's largest patient cohort is of low acuity, thus requiring fewer binding resources per patient.

The use of electronic and manual platforms led to exclusions; gaining a full sample was reliant on the data points matching up between the platforms. Data points that did not exist in both electronic and manual data sets, as well as duplicate records or records with missing data points, were removed. However, early reports suggested that for cases where manual records were present but were not reflect electronically, these patients were streamed to outpatient departments and not seen in the EC. For cases where electronic records were present and not reflected manually, operator omission was considered, or the EC operations required a bypass of triage for unknown reasons. It is unlikely that these missing data points would have affected the results of this study. It was found and acknowledged that a large portion of children's records was excluded due to missing blood pressure entries.

Evaluating timeframes were complex with most of the time stamps requiring manual entry and those that were self‐generated by the hospital information system still were at the mercy of staff entry time. Incorrect times could have resulted from unsynchronized clocks, forgetting to accurately determine and record the times, or making late entries on the hospital information system. It was accepted that some variation of time records existed as this was dependant on human input. Using the medians as a measure of central tendency helped mitigate any absolute outliers that could have influenced the findings.

## 6 CONCLUSION

The purpose of triage is to match the correct available resources to patient needs when presenting to an EC. Triage category allocations and their associated timeframes attempt to structure patients in such a way that the most ill or injured patients are attended to first. This study has shown that in the private healthcare setting of this hospital group that patients of all acuities are attended to in a relatively short timeframe. These timeframes even exceed the expectations of the guidelines put forward by the triage systems in use. By shortening the time patients wait to see a physician and get appropriate treatment, it will not only improve morbidity and mortality, but also improve the patient experience. These benchmarks would greatly assist this and other private hospital groups in the region to set targets for their own triage system.

## 7 RELEVANCE TO CLINICAL PRACTICE

Timely care in any healthcare setting is crucial for the effective management of a patients' illness or injury. Mortality and morbidity have shown to decrease when treatment is given sooner rather than later. There is an increasing patient population that presents to ECs, increasing the strain on available resources. To ensure the most critically ill or injured patients are attended to first, it is crucial for a triage system to distinguish acuity accurately and consistently. Once a patient is triaged, it is vital for them to be attended to within the relative timeframes associated with their acuity, to ensure timely emergency care is provided. Evaluating the performance of an EC to meet these targets (and to make changes where necessary) strengthens the clinical ability of the unit to manage patients effectively.

## CONFLICT OF INTEREST

This study was conducted in the emergency centres of Mediclinic Middle East who highlighted triage as a vital component of their business model. The author declares no conflict of interest beyond the support provided by the management of Mediclinic Middle East to undertake this study.

## References

[nop2336-bib-0001] Beveridge, R. , Clarke, B. , Janes, L. , Savage, N. , Thompson, J. , Dodd, G. , … Vadeboncoeur, A. (1998). Implementation Guidelines for the Canadian Emergency Department Triage & Acuity Scale (CTAS). Canadian Association of Emergency Physicians, pp. 1–32.

[nop2336-bib-0012] Cheema B., & Twomey M. (Eds.) (2012), The South African Triage Scale (SATS) (pp. 1–35). Cape Town, South Africa : South African Triage Group.

[nop2336-bib-0002] Dippenaar, E. (2016). Standardisation and validation of a triage system in a private hospital group in the United Arab Emirates. University of Cape Town. Retrieved from http://open.uct.ac.za/bitstream/handle/11427/23397/thesis_hsf_2016_dippenaar_enrico.pdf?sequence=1

[nop2336-bib-0003] Dubai Health Authority [Internet] (2019). Retrieved from https://www.dha.gov.ae/EN/Pages/default.aspx

[nop2336-bib-0004] Fry, M. , & Burr, G. (2002). Review of the triage literature: Past, present, future? Australian Emergency Nursing Journal, 5(2), 33–38. 10.1016/S1328-2743(02)80018-9

[nop2336-bib-0005] Gilboy, N. , Tanabe, T. , Travers, D. , & Rosenau, A. M. (2011). Emergency Severity Index (ESI): A triage tool for emergency department care, Version 4. Implementation Handbook 2012 Edition. (AHRQ Publication No. 12-0014). Rockville, MD: Agency for Healthcare Research and Quality..

[nop2336-bib-0006] Gravel, J. , Fitzpatrick, E. , Gouin, S. , Millar, K. , Curtis, S. , Joubert, G. , … Osmond, M. H. (2013). Performance of the canadian triage and acuity scale for children: A multicenter database study. Annals of Emergency Medicine, 61(1), 27–32. 10.1016/j.annemergmed.2012.05.024 22841173

[nop2336-bib-0007] Health Authority – Abu Dhabi [Internet] (2019). Retrieved from http://www.haad.ae/haad/

[nop2336-bib-0008] Johnson, L. A. (1996). Correspondence – Triage: Limitations and opportunities. Annals of Emergency Medicine, 28(3), 372–374. 8780492 10.1016/s0196-0644(96)70044-6

[nop2336-bib-0009] Kennedy, K. , Aghababian, R. , Gans, L. , & Lewis, C. (1996). Triage: Techniques and applications in decisionmaking. Annals of Emergency Medicine, 28(2), 136–144. 10.1016/S0196-0644(96)70053-7 8759576

[nop2336-bib-0010] Mackway‐Jones K., Marsden J., & Windle J. (Eds.) (2006). Emergency triage (Chapters 1 to 3), Manchester Triage Group (2nd ed., pp. 1–171). Hoboken, NJ: Blackwell Publishing.

[nop2336-bib-0011] Parenti, N. , Manfredi, R. , Bacchi Reggiani, M. L. , Sangiorgi, D. , & Lenzi, T. (2010). Reliability and validity of an Italian four‐level emergency triage system. Emergency Medical Journal, 27(7), 495–498. 10.1136/emj.2008.070193 20584948

[nop2336-bib-0013] Van der Linden, C. , Lindeboom, R. V. , der Linden, N. , & Lucas, C. (2012). Managing patient flow with triage streaming to identify patients for Dutch emergency nurse practitioners. International Emergency Nursing, 20(2), 52–57. 10.1016/j.ienj.2011.06.001 22482999

[nop2336-bib-0014] van der Wulp, I. (2010). Reliability and validity of emergency department triage systems (pp. 1–144). Enschede, the Netherlands: Gildeprint Drukkerijen.

[nop2336-bib-0015] von Elm, E. , Altman, D. G. , Egger, M. , Pocock, S. J. , Gøtzsche, P. C. , & Vandenbroucke, J. P. (2008). The Strengthening the Reporting of Observational Studies in Epidemiology (STROBE) statement: Guidelines for reporting observational studies. Journal of Clinical Epidemiology, 61(4), 344–349. 10.1016/j.jclinepi.2007.11.008 18313558

